# *Pseudomonas putida* infection induces immune-antioxidant, hepato-renal, ethological, and histopathological/immunohistochemical disruptions in *Oreochromis niloticus:* the palliative role of titanium dioxide nanogel

**DOI:** 10.1186/s12917-024-03972-6

**Published:** 2024-04-01

**Authors:** Afaf N. Abdel Rahman, Heba H. Mahboub, Gehad E. Elshopakey, Mahmoud I. M. Darwish, Heba Said Abdel-Rahman Gharib, Mohamed Shaalan, Esraa M. Fahmy, Heba M. Abdel-Ghany, Sameh H. Ismail, Hassnaa Mahmoud Elsheshtawy

**Affiliations:** 1 Department of Aquatic Animal Medicine, Faculty of Veterinary Medicine, Zagazig University, PO Box 44511, Zagazig, Sharkia Egypt; 2 Department of Clinical Pathology, Faculty of Veterinary Medicine, Mansoura University, PO Box 35516, Mansoura, Dakahlia Egypt; 3 Department of Biochemistry and Molecular Biology, Faculty of Veterinary Medicine, Zagazig University, Zagazig, PO Box 44511, Sharkia Egypt; 4 Department of Behaviour and Management of Animal, Poultry, and Aquatics, Faculty of Veterinary Medicine, Zagazig University, PO Box 44511, Zagazig, Sharkia Egypt; 5 Department of Pathology, Faculty of Veterinary Medicine, Cairo University, PO Box 12211, Giza, Egypt; 6 Polymer Institute, Slovak academy of sciences, Dúbravská cesta 9, 845 41, Bratislava, Slovakia; 7 Department of Pharmacology, Faculty of Veterinary Medicine, Zagazig University, PO Box 44511, Zagazig, Sharkia Egypt; 8 Department of Pathology, Faculty of Veterinary Medicine, Zagazig University, PO Box 44511, Zagazig, Sharkia Egypt; 9 Faculty of Nanotechnology for Postgraduate Studies, Cairo University, Sheikh Zayed Branch Campus, PO Box 12588, Sheikh Zayed City, Giza Egypt; 10 Department of Fish Diseases and Management, Faculty of Veterinary Medicine, Suez Canal University, PO Box 41522, Ismailia, Egypt

**Keywords:** Bacterial infection, Health status, Nile tilapia, *Pseudomonas putida*, Immune response, Titanium dioxide nanogel

## Abstract

**Background:**

*Pseudomonas putida* is a pathogenic bacterium that induces great losses in fishes, including Nile tilapia (*Oreochromis niloticus*). Currently, the application of nanomaterials in aquaculture practices has gained more success as it endows promising results in therapies compared to traditional protocols.

**Objective:**

Therefore, the current perspective is considered the first report to assess the anti-bacterial efficacy of titanium dioxide nanogel (TDNG) against *Pseudomonas putida *(*P. putida*) in Nile tilapia.

**Methods:**

The fish (*n* = 200; average body weight: 47.50±1.32 g) were allocated into four random groups (control, TDNG, *P. putida*, and TDNG + *P. putida*), where 0.9 mg/L of TDNG was applied as bath treatment for ten days.

**Results:**

Outcomes revealed that *P. putida* infection caused ethological alterations (surfacing, abnormal movement, and aggression) and depression of immune-antioxidant variables (complement 3, lysozyme activity, total antioxidant capacity, superoxide dismutase, and reduced glutathione content). Additionally, a substantial elevation in hepatorenal biomarkers (aspartate and alanine aminotransferases and creatinine) with clear histopathological changes and immuno-histochemical alterations (very weak BCL-2 and potent caspase-3 immuno-expressions) were seen. Surprisingly, treating *P. putida-*infected fish with TDNG improved these variables and obvious restoration of the tissue architectures.

**Conclusion:**

Overall, this report encompasses the key role of TDNG as an anti-bacterial agent for controlling *P. putida* infection and improving the health status of Nile tilapia.

**Supplementary Information:**

The online version contains supplementary material available at 10.1186/s12917-024-03972-6.

## Introduction

In aquaculture, the tilapia industry is one of the most stable and steady-growing. Nile tilapia (*Oreochromis niloticus*) is the best-ranked and most substantial tilapia species being cultivated and traded [1]. With rapid progress and intensification in the aquaculture industry, various emerging diseases have arisen [2]. In particular, bacterial infections induce hazardous effects on fish, including septicemia, hemorrhage, and mortalities [3, 4].

Among bacterial diseases, the *Pseudomonas species* is one of the most virulent pathogens that invades fish and results in ulcerative syndrome [5]. *Pseudomonas putida* is a highly pathogenic bacterium that infects Nile tilapia and induces ascites, exophthalmia, and ulcers in the body [6]. *P. putida* is an opportunistic Gram-negative pathogen related to the *Pseudomonaceae* family that normally exists in the aquatic ecosystem. It is found in healthy fish as a part of the normal gut microflora [7, 8]. A recent study reports that *P. putida* causes higher mortalities in Nile tilapia as it carries virulence-linked genes (ToxA, Nan1, and ExoS) [9].

The emergence of drug-resistant bacteria increases the demand for alternative strategies to treat bacterial infections. One of the successful strategies is utilizing nano-based materials as anti-bacterial agents [10, 11]. The nanomaterials are utilized as antimicrobial agents because of their power to penetrate the membranes of the bacteria and disrupt the formation of biofilm [12]. In particular, metallic nanoparticles (NPs) are attracting more attention owing to their great success in pharmaceutical and biological applications [13, 14]. Various metal oxide-NPs exhibited potent antimicrobial activity, like titanium, magnesium, zinc oxide, copper, and silicon oxides. They are characterized by many features, including heat resistance and less toxicity, and reveal strong activity against resistant strains of many microorganisms [15, 16]. Also, they can be utilized as mineral element supplements which are essential for nourishing cells [17]. Nano-based titanium oxide elicited potent inhibitory activity against the growth of bacteria because of its little nano-sized and strong oxidizing activity [18].

Nanogels (NGs) are a recent and superior scheme for diagnosing and treating a broad spectrum of diseases [19]. Due to their small size, NGs assist as significant drug nano-carriers, which can penetrate tissues in a transcellular way [20]. The gel-derived titanium dioxide (TiO_2_) has a tested anti-bacterial activity [21]. Recently, it has proved the effective inhibitory activity of TiO_2_ nano-form against 15 species of bacteria [22]. Current studies apply other types of NGs in aquaculture practice and prove great success in alleviating toxicity and promoting immune-antioxidant function [23, 24].

Hence, the present report is a pioneering trial to investigate the anti-bacterial impact of TiO_2_ nanogel (TDNG) as a watery addition on the health status of Nile tilapia challenged with* P. putida.* The investigation included assaying the fish behavior, immunity, antioxidant activity, and histological architectures. This gives a theoretical basis for the logical application of TDNG.

## Materials and methods

### TDNG synthesis and characterization

Firstly, the synthesis of titanium dioxide nanoparticles (TiO_2_NPs) was achieved through a simple sono-chemical method [25]. 0.25 g of TiO_2_ (Sigma-Aldrich Co., MO, USA) was added to 100 mL of 10 M NaOH (El Naser Chemical Co., Egypt) in a 250 mL flask. The solution was then subjected to ultrasonic waves (Sonica 4200 EPS3, Milano, Italy) under the condition of 88% amplitude and 0.82 cycles for 1.5 h at room temperature with adjusting pH to 7.0 using 0.1 M HCL (El Naser Chemical Co., Egypt). The resulting TiO_2_NPs solution was then centrifuged three times using double distilled deionized water for washing.

To synthesize TDNG as TiO_2_ NPs/carbopol hybrid nanogel, 0.2 g of TiO_2_NPs was dispersed in 40 mL of ethanol (95%) and added to 0.4 g of carbopol dissolved in 40 mL of ethanol (95%). The resulting mixture was stirred using a mechanical stirrer for 65 min. Then, 1.2 mL of trimethylamine was added drop by drop, and the stirring continued for another 65 min. until a white gel was obtained. TDNG was prepared in low and high-viscosity forms. Characterization procedures were divided into three categories: morphology, index, and identification [26].

### Acclimation of fish and ethical approval

The experimental design of the current study was approved by the Institutional Animal Care and Use Committee at Zagazig University in Egypt (ZU-IACUC/2/F/333/2022). Nile tilapia (47.50 ±1.32 g) were sorted from a private fish farm in Al-Abbassa, Sharkia Governorate, Egypt with prior informed consent from the owners. For acclimation, fish were maintained for 2 weeks in 100 L of well-aerated aquaria (ten fish/ aquarium). The excretory wastes were disposed of daily through siphoning. The fish received a commercial diet at 3% of their body mass twice daily throughout the acclimation period. The physio-chemical biomarkers (temperature, dissolved oxygen, ammonia, and pH) of the rearing water were monitored daily according to APHA [27] guidelines, and they were 23.00±2 °C, 6.50 ± 0.11 mg/L, 0.01 ± 0.03 mg/L, and 7.30 ± 0.12, respectively.

### Assessing the initial concentration of TDNG

Fish (*n* = 70) were exposed to 7 different concentrations (0.0, 0.3, 0.6, 0.9, 1.2, 1.5, and 1.8 mg/L) of TDNG for ten days to establish the initial concentration for the treatment trial. Each group was kept in 100 L well-aereated aquarium (10 fish/aquarium). Weighed amounts of TDNG was dissolved in initial amount of distilled water (5 mL) before adding to the aquarium water to obtain the final concentrations. Daily records of the clinical observations were kept throughout the initial study. TDNG concentrations were deemed safe between 0.3 and 1.2 mg/L, with 0.9 mg/L being the concentration employed for treatment (Supplementary Table 1).

### Bacterial strain *(P. putida*)

The current perspective was carried out on *P. putida*, formerly isolated from diseased Nile tilapia (Department of Aquatic Animal Medicine, Faculty of Veterinary Medicine, Zagazig University). It was recognized by the VITEK 2-C15 automated system for bacterial identification (BioMérieux, Craponne France) and conventional biochemical assays following the manufacturer’s instructions as documented by Scheidegger et al. [28] and Zhou et al. [29] at the Department of Microbiology and Immunology, National Research Centre (NRC), Dokki, Giza, Egypt. *P. putida* was streaked onto pseudomonas agar base (Oxoid, England) and incubated for 24 h at 37 °C. One colony was taken to incubate in brain heart infusion broth (Sigma-Aldrich) for 24 h at 37 °C. After centrifuging the cultured broth at 4 °C for 10 min at 3000 ×g, the pellet was retrieved and suspended in sterile phosphate-buffered saline (PBS).

The lethal dose (LD_50_) of *P. putida* was established. Fish (*n*=80) were distributed into four groups in duplicates (10 fish/ replicate; 20 fish/group). Fish were given intraperitoneally (IP) different doses of a live, 24 h-old *P. putida* culture suspension (10^6^– 10^9^ CFU/fish). Another ten fish (control group) were IP given with 0.1 mL sterile saline. The mortality of fish was then noted four days following the inoculation. According to the Probit Analysis Program, version 1.5 (US Environmental Protection Agency), the LD_50_ was 3.9 × 10^8^ CFU/mL. A sub-lethal dose of 1.5 × 10^8^ CFU/mL was used in the trial.

### Experimental protocol

For ten days, fish (*n* = 200) were randomized into four groups in five replicates (10 fish/replicate). The groups were the control (no TDNG addition or *P. putida* challenge), TDNG, *P. putida*, and TDNG + *P. putida* groups. The fish of *P. putida* and TDNG + *P. putida* groups were IP injected with 0.1 mL of *P. putida* (1.5 ×10^8^ CFU/mL). After the onset of clinical signs, 0.9 mg/L of TDNG was added to the aquarium water, and this procedure was continued for ten days. The excretory wastes were disposed daily through siphoning and complete water exchange was performed three times weekly. To maintain the applied TDNG concentration after water exchange, the freshly made TDNG solution was added. During the trial's ten-day run, daily records of clinical signs and mortality were kept.

### Behavioural investigations

The behavior patterns were recorded in all experimental groups at a fixed time once daily (8-9 a.m.) throughout the experimental period by direct observation technique using a video camera and stopwatch [30]. The behavior frequencies were observed for 15 min. intervals (5 min/aquarium). The recorded behavior categories were foraging, swimming [31], surfacing [32], resting [33], and abnormal movement [34]. Moreover, aggressive behaviour was recorded, including approach, chasing, fleeing, fin tugging, butting, and mouth pushing [35].

### Sampling

On the last day of the ten-day experiment, fish (15 fish per group) were randomly picked to drain samples. Based on Neiffer and Stamper [36] method, fish were anesthetized using a benzocaine solution (100 mg/L), and then blood was collected from the caudal blood vessels using tubes without anticoagulant. Centrifugation was carried out for the samples at 1750 *×* g for 10 min after incubation at 22±2 °C for 5 h. Pure serum was then maintained at 20 °C to assess biochemical and immunological parameters. Hepatic and renal samples were collected for antioxidant, histopathological, and immunohistochemical assessments.

### Biochemical and immunological assays

The serum levels of liver function parameters involving aspartate aminotransferase (AST, Catalog No.; AS1061) and alanine aminotransferase (ALT, Catalog No.; AL 1031) and kidney biomarkers, including creatinine (Catalog No.: CR 1250) (Biodiagnostic Co., Egypt) were estimated. All the above-recorded biomarkers were recorded using a spectrophotometer (Lambda EZ201; Perkin Elm, Beaconsfield, UK).

The immune biomarkers in serum, including complement 3 (C3) and lysozyme (LYZ) activities were assessed. Both of them play a substantial role in the innate immune function of fish against infection. The C3 was determined by immuno-turbidimetry using Cusabio kits (CUBIO Innovation Center, Houston, USA) with a Catalog No (CSB-E09727s) following the manufacturer protocol. Meanwhile, the level of LYZ was measured using the inhibition zone method in agarose gel plates [37].

### Antioxidant assays

Hepatopancreas samples were homogenized in 10% w/v PBS (pH 7.4) of Sigma-Aldrich (St. Louis, MO, USA) before centrifugation at 10,000 *×* g for 20 min (4 °C). Following that, the supernatant was centrifuged at 10,000 *×* g for 1 hour (4 °C). The level of total antioxidant capacity (TAC), superoxide dismutase (SOD), and reduced glutathione content (GSH) were estimated in hepatopancreas tissues following the protocols of Koracevic et al. [38], Velkova-Jordanoska et al. [39], and Beutler et al. [40].

### Histopathological and immunohistochemical investigation

Hepatopancreas and kidney samples were collected from all experimental groups, fixed using 10% buffered neutral formalin, then dehydrated using ascending degrees of alcohol, cleared in xylene, and finally soaked in paraffin. Paraffin sections (5 μm in thickness) were cut and stained using hematoxylin and eosin (H&E) for general histology and then inspected by an optical microscope fitted with camera system (Olympus BX53), following a prior method [41].

The immunohistochemical (IHC) assay was conducted in the de-paraffinized section (5 μm thick) with an in situ cell apoptosis identification kit (MK1020, Boster, China) according to the manufacturer’s guidelines, as reported [42]. B-cell lymphoma 2 (BCL-2) and caspase-3 were identified by IHC. For this reason, concisely, an HRP/DAB detection IHC kit (ab80436 Abcam, China) was utilized based on the manufacturer’s method. The sections were exposed to de-paraffinization, and then, sections carrying formalin-fixed paraffin tissues were rehydrated. A block of hydrogen peroxide was included to protect the sections, then incubated for 10 min. Following antigen retrieval (100 × Citrate Buffer, ab64236 Abcam, China) for 20 min, the sections were exposed to immunoreaction over-night at 4 °C using 10 μg/mL primary antibodies (AB- 20074b and AB20158b, Sangon Biotech, China) counter to BCL-2 and caspase-3 in case of negative controls, the sections were dipped in PBS as a substitute of the definite antibody. Then, mouse-specific HRP was used for conjugation and incubation for 15 min at room temperature. DAB was used for the tissue sections that were counterstained using hematoxylin.

### Analysis of data

Via Shapiro–Wilk normality, all collected data were examined for norm homogeneity. Following that, the data were statistically analyzed using one-way-ANOVA (analysis of variance test) using SPSS version 22 (SPSS, Richmond, VA, USA). Tukey's range test was conducted to evaluate the differences between means at a 95% confidence level. The means ± standard error (*SE*) were used to present the data. To investigate the survival probability of fish in each group, the Kaplan-Meier model was applied. Moreover, the Mantel-Cox (log-rank) test was applied to see if there were any variations between the groups.

## Results

### Characterization of TDNG

**Fig. 1:**
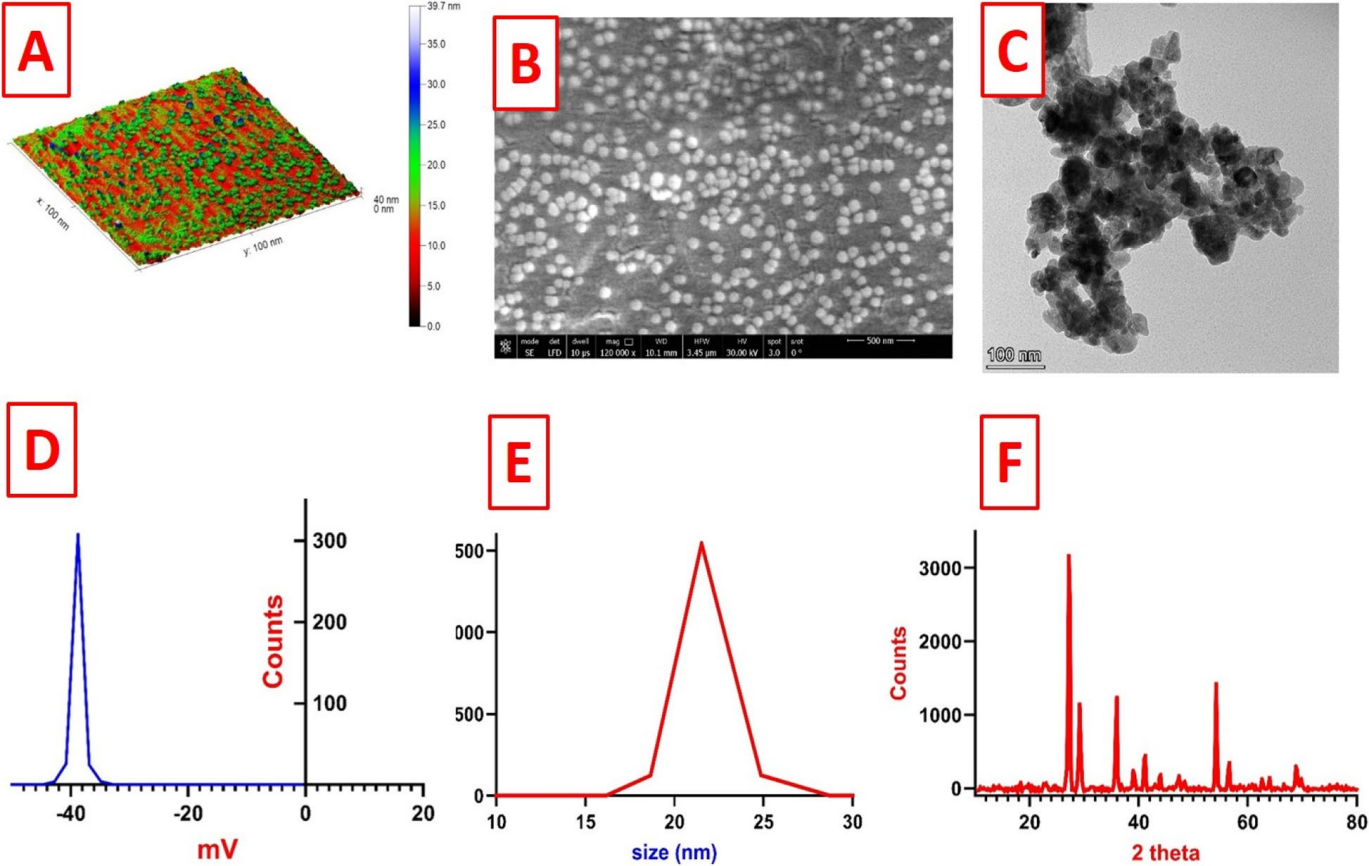
The characterization images of TDNG. **A** AFM. **B** SEM (500 nm). **C** TEM (100 nm). **D** zeta potential. **E** DLS. **F** XRD

Figure 1 : displays different classes of TDNG characterization. The morphology class included surface morphology and particle shape that was retrieved in atomic force microscopy (AFM; Fig. 1A), scanning electron microscopy (SEM; Fig. 1B), and transmission electron microscopy (TEM; Fig. 1C) images. They exhibited that the TDNG had a spherical shape. The index class (size and surface charge) presented in zeta potential (Fig. 1D) and dynamic light scattering images (DLS; Fig. 1E). The data revealed that the particles had a very good colloidal nature in aqueous solution, as indicated by the high zeta potential value of -41mV. Also, the particles were homogeneous in size (with only one peak in the DLS chart), with a size of approximately 22 nm.

The identification class appeared in the X-ray diffraction image (XRD; Fig. 1F). It verified that no secondary phases were necessary for the synthesis process to be valid. However, there were no distinctive peaks because the gel creation was amorphous.

### Behavioral alterations

Table 1 shows the different behaviors recorded during the trial (ten days). There were no behavioral alterations recorded in the control and TDNG groups.* P. putida* infection caused a significant reduction in foraging (*p*<0.05) and an increase in the abnormal swimming, surfacing, resting, aggression traits (approach, chasing, fleeing, and mouth pushing), and abnormal movement (circular and vertical) as compared with control. On the contrary, treatment of infected with TDNG (TDNG + *P. putida* group) markedly enhanced* (p*<0.05) the foraging and declined the other behaviors compared with the infected one without treatment (*P. putida* group)*.* The other aggressive traits (fin tugging and butting) did not significantly alter between groups.

**Table 1:** The behavioral alterations of Nile tilapia experimentally infected with *Pseudomonas putida* and exposed to titanium dioxide nanogel (TDNG) (0.9 mg/L) for ten days

Parameters	Control	TDNG	*P. putida*	TDNG+*P. putida*	*P-*value
Foraging	1.50±0.22^a^	1.30±0.23^a^	0.33±0.16^c^	0.77±0.44^b^	0.004
Abnormal swimming	2.16±0.30^c^	2.55±0.44^c^	10.11±1.11^a^	8.33±0.55^b^	<0.0001
Surfacing	0.50±0.22^c^	0.70±0.28^c^	3.00±057^a^	2.33±0.60^b^	0.005
Resting	1.50±0.22^c^	1.11±0.20^c^	2.33±0.47^a^	1.88±0.30^b^	0.073
**Aggressive behaviour**					
Approach	0.66±0.21^c^	0.88±0.20^c^	3.33±0.57^a^	1.66±0.44^b^	< 0.0001
Chasing	0.16±0.16^c^	0.22±0.27^c^	2.55±0.47^a^	1.66±0.16^b^	< 0.0001
Fleeing	0.66±0.33^c^	0.73±0.47^c^	3.22±0.74^a^	2.33±0.28^b^	0.012
Fin tugging	0.50±0.34	0.55±0.17	1.3±0.33	0.77±0.22	0.13
Butting	0.00±00	0.00±00	0.22±0.14	0.11±0.11	0.35
Mouth pushing	0.00±00^c^	0.00±0.27^c^	1.44±0.44^a^	1.00±0.23^b^	0.04
**Abnormal movement**					
Circular movement	0.16±0.16^c^	0.21±0.26^c^	2.00±0.55^a^	1.77±0.40^b^	0.027
Vertical movement	0.00±00^c^	0.00±0.24^c^	2.11±0.51^a^	0.88±0.11^b^	0.001

Values (means± SE) in the same row that do not share the same superscripts differ substantially (*p* < 0.05)

### Clinical observations and survival rate

**Fig. 2:**
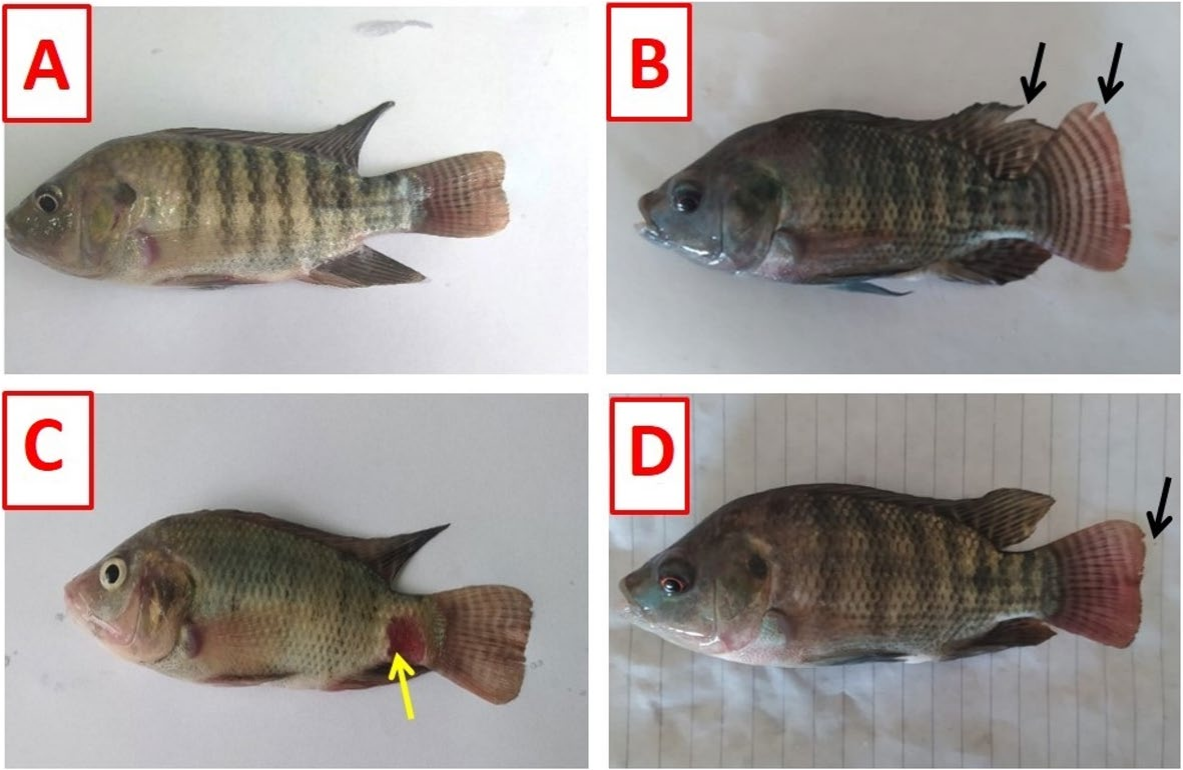
The clinical signs of Nile tilapia experimentally infected with* Pseudomonas putida* and exposed to titanium dioxide nanogel (TDNG) (0.9 mg/L) for ten days. **A** Control and TDNG fish display a normal appearance. **B** & **C** Fish of the *P. putida* group display skin darkness, body hemorrhages, fin rot (black arrows), and severe skin ulcerations (yellow arrow). **D** Fish of the TDNG + *P. putida* group displaying skin darkness, redness of the caudal fin, and fin rot (black arrow)

**Fig. 3:**
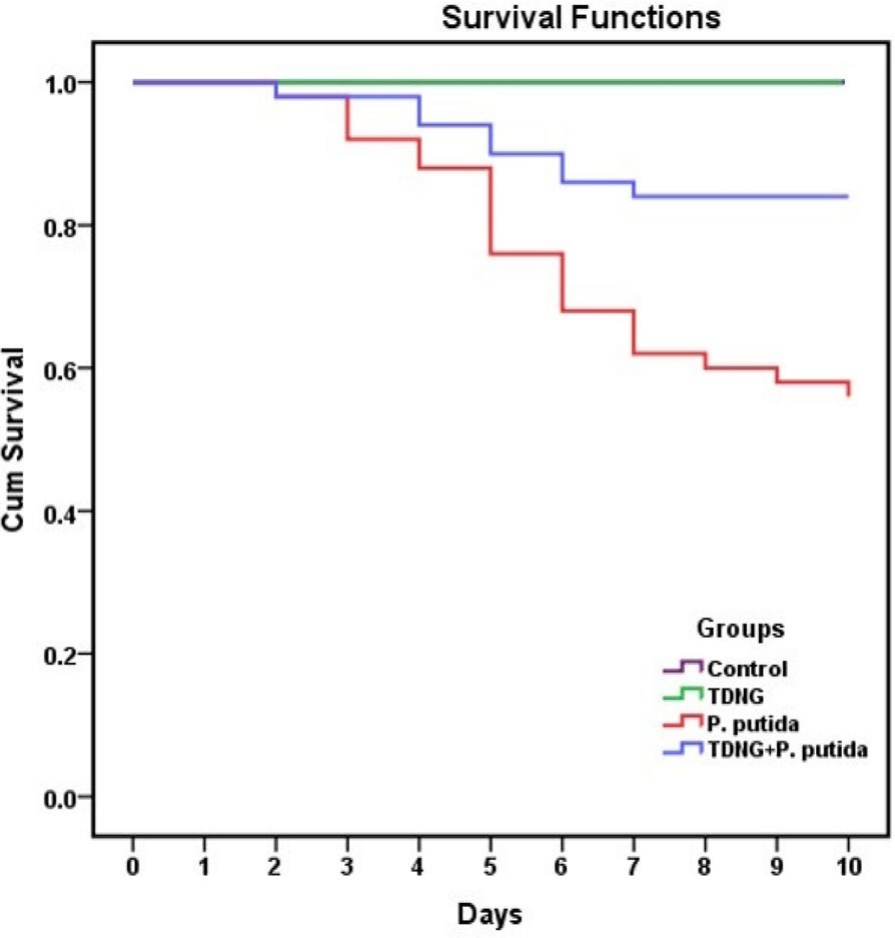
The Survival curves of Nile tilapia experimentally infected with *Pseudomonas putida* and exposed to titanium dioxide nanogel (TDNG) (0.9 mg/L) for ten days

Figure 2A displays no obvious clinical signs in the control and TDNG groups. *P. putida* infection induced various clinical signs, including skin darkness, body hemorrhages, fin rot, and severe skin ulcerations (Fig. 2B and C). Contrarily, administration of 0.9 mg/L TDNG to infected fish (TDNG + *P. putida*) improved the previous clinical signs. However, some fish showed skin darkness and redness of the caudal fin with fin rot (Fig. 2D). According to the Kaplan-Meier curves (Fig. 3), the survival rate in the control, TDNG,* P. putida,* and TDNG + *P. putida* groups was 100, 100, 56%, and 84%, respectively. Additionally, there were group-specific statistically significant differences (*p*<0.0001).

### Biochemical variables

**Fig. 4:**
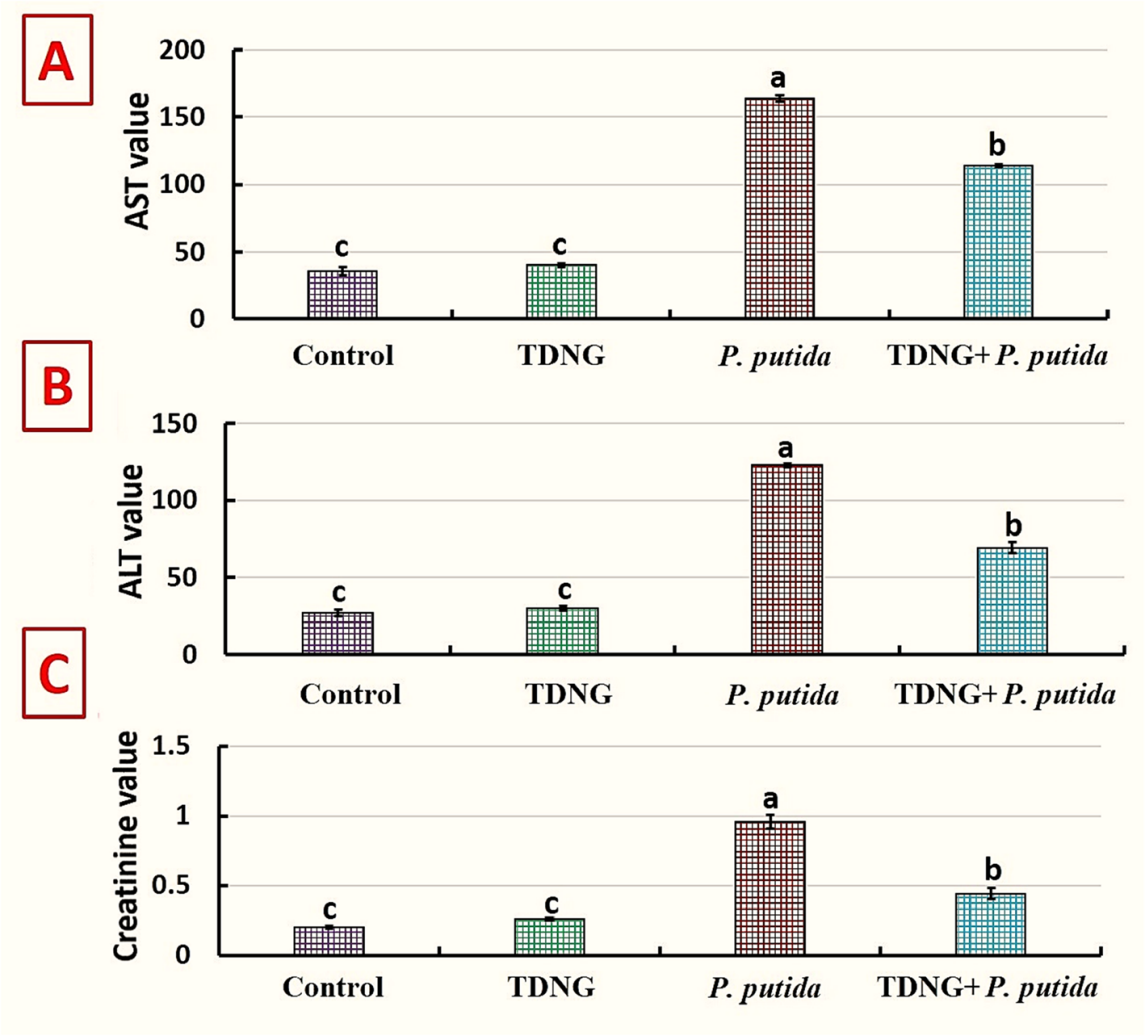
The biochemical variables of Nile tilapia experimentally infected with *Pseudomonas putida* and exposed to titanium dioxide nanogel (TDNG) (0.9 mg/L) for ten days. **A** Aspartate aminotransferase (AST; U/mL, *p*<0.0001). **B** Alanine aminotransferase (ALT; U/mL, *p*<0.0001). **C** Creatinine (mg/dL, *p*<0.0001)

Between the control and TDNG groups, there are no discernible variations in the values of AST, ALT, or creatinine (*p* > 0.05), according to Fig. 4. The *P. putida* group showed a considerable increase (*p<*0.0001) in these variables when compared to the control. The TDNG + *P. putida* group showed a markedly significant drop (*p<*0.0001) in these variables compared to the *P. putida* group.

### Immune- antioxidant variables

As shown in Table 2, the level of C3 and LYZ (immunological indicators) and TAC, SOD, and GSH (antioxidant markers) in the TDNG group markedly increased (*p<*0.0001) compared with control. These variables of the *P. putida* group showed a significant decrease (*p<*0.0001) relative to the control. In contrast to the *P. putida* group, there was a significant improvement (*p<*0.0001) in these markers in the treated group (TDNG + *P. putida*).

**Table 2:** The immune-antioxidant variables of Nile tilapia experimentally infected with *Pseudomonas putida* and exposed to titanium dioxide nanogel (TDNG) (0.9 mg/L) for ten days

Parameters	Control	TDNG	*P. putida*	TDNG+*P. putida*	*P-*value
**Serum immune variables**					
C3 (μg/mL)	35.40±1.97^b^	46.18±1.47^a^	12.00±1.63^d^	25.04±1.15^c^	< 0.0001
LYZ (ng/ mL)	1.85±0.04^b^	2.54±0.26^a^	0.30±0.05^d^	0.89±0.04^c^	< 0.0001
**Hepatic antioxidant variables**					
TAC (ng/mg tissue)	8.79±0.41^b^	15.83±1.18^a^	3.25±0.18^d^	5.64±0.66^c^	< 0.0001
SOD (U/mg tissue)	123.53±2.39 ^b^	180.43±5.62^a^	47.41±1.22^d^	70.78±2.81^c^	< 0.0001
GSH (ng/mg tissue)	133.80±2.25^b^	210.80±3.29^a^	62.37±1.44^d^	76.85±1.07^c^	< 0.0001

C3: complement 3; LYZ: lysozyme; TAC: total antioxidant capacity; SOD: superoxide dismutase; GSH: reduced glutathione content. Values (means± SE) in the same row that do not share the same superscripts differ substantially (*p* < 0.05)

### Histopathological findings

**Fig. 5:**
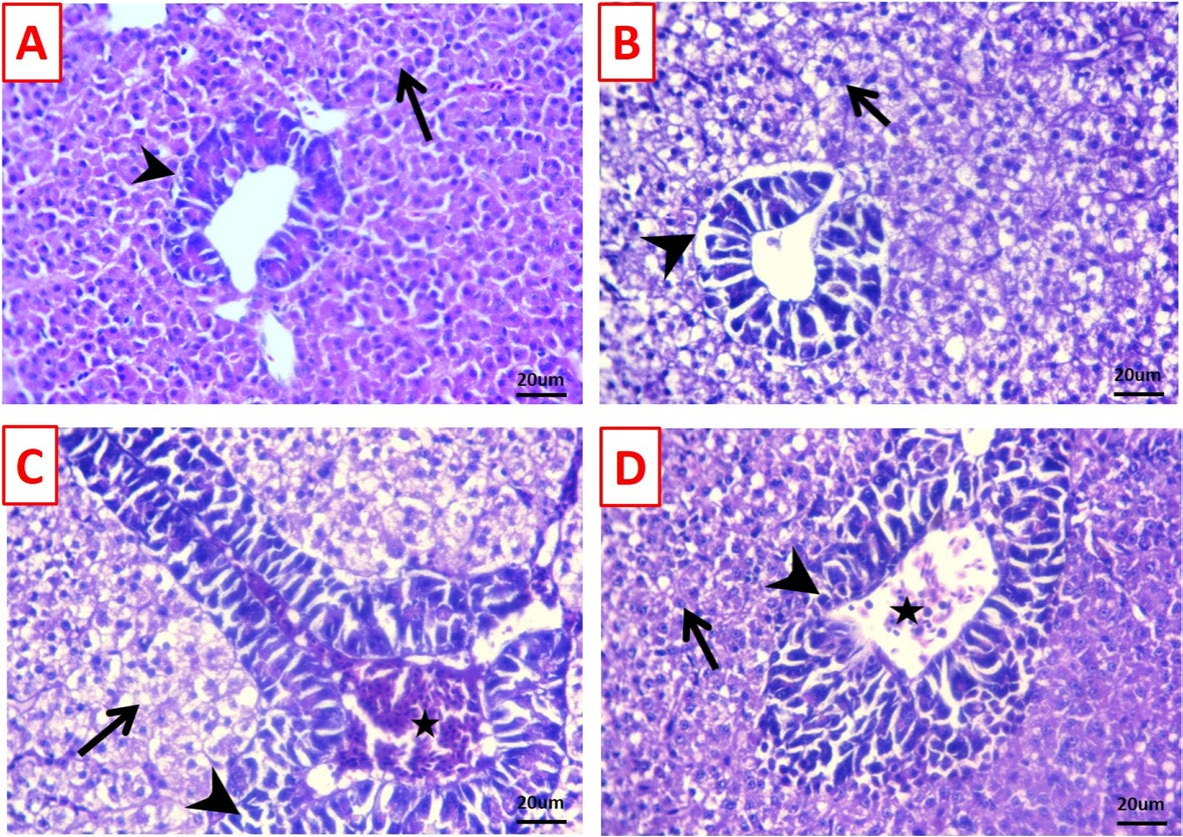
Photomicrographs of stained hepato-pancreas sections (H&E; scale bar 20μm). **A** Hepato-pancreas of the control group displaying normal histo-architectures of hepatic cells (arrow) and pancreatic acini (arrowhead). **B** Hepato-pancreas of the titanium dioxide nanogel (TDNG) group displays vacuolated hepatocytes (arrow) and preserved pancreatic acini (arrowhead). **C** The hepato-pancreas of the *Pseudomonas putida* group displays focal areas of degenerated and necrotic hepatocytes (arrow), a few apoptotic areas of pancreatic acini (arrowhead), and a congested portal vein (star). **D** Hepato-pancreas of the TDNG + *P. putida* group displays a dilated portal vein (star) with maintaining architectures of hepatocytes (arrow) and pancreatic acini (arrowhead)

The hepato-pancreas of the control group showed normal histo-architectures of hepatic cells and pancreatic acini (Fig. 5A). As well, the hepatic parenchyma of the TDNG group were vacuolated due to fat or glycogen storage beside preserved pancreatic acini (Fig. 5B). While, focal areas of degenerated and necrotic hepatocytes, few apoptotic areas of pancreatic acini, and congested portal vein were the most encountered lesions observed in the hepato-pancreas of *P. putida* group (Fig. 5C). These alterations were decreased in the TDNG + *P. putida* group, where dilated portal vein with maintain architectures of hepatocytes and pancreatic acini were demonstrated (Fig. 5D).

**Fig. 6:**
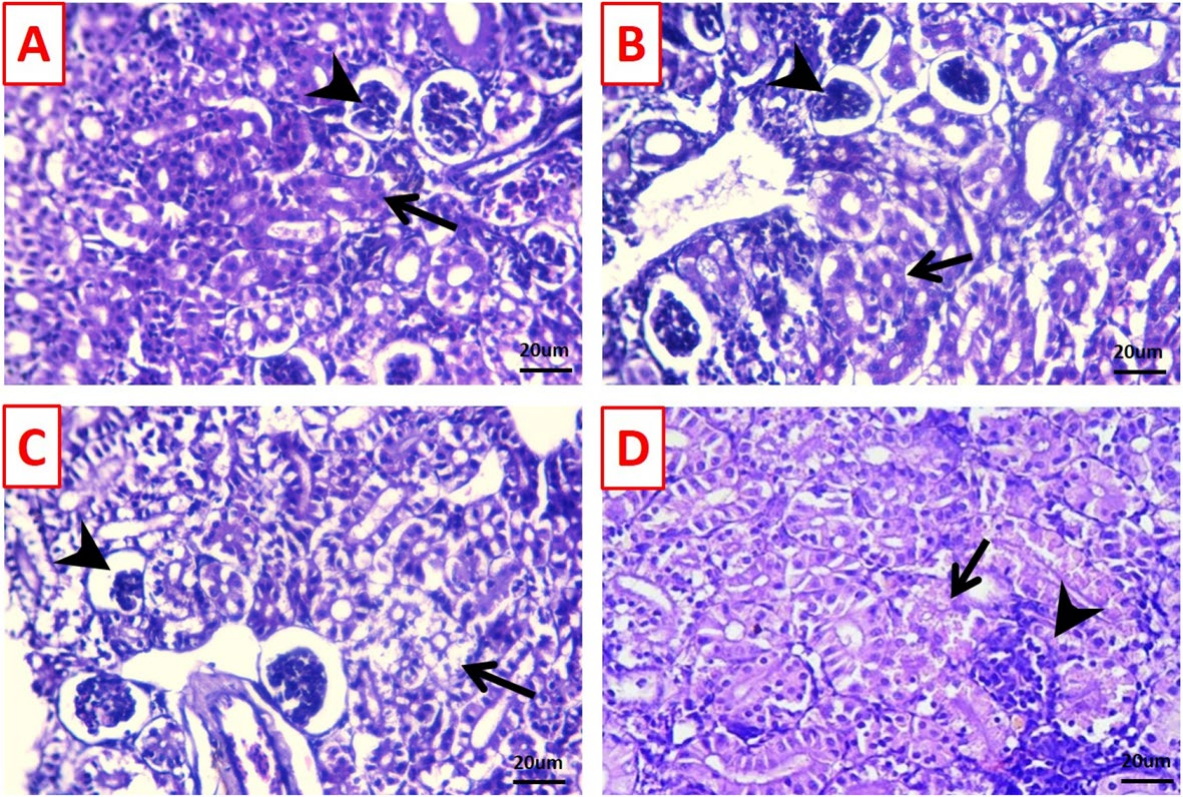
Photomicrographs of stained kidney sections (H&E; scale bar 20μm). **A** & **B** Kidneys of the control and titanium dioxide nanogel (TDNG) groups display normal cytoarchitecture of glomerular tufts (arrowheads) and renal tubules (arrows). **C** The kidney of the *Pseudomonas putida* group displays a necrotic tubule (arrow) and some atrophied glomeruli (arrowhead). **D** The kidney of the TDNG + *P. putida* group displays hyaline globules within a few numbers of the renal epithelium (arrow) and apparent normal renal parenchyma and glomerular structures (arrowhead)

Moreover, the kidneys of the control and TDNG groups displayed normal cytoarchitecture of glomerular tufts and renal tubules (Fig. 6A and B). While *P. putida* induced (Fig. 6C) the presence of necrotic tubules and some atrophied glomeruli. In addition, the kidney of the TDNG + *P. putida* group exhibited hyaline globules within a few numbers of the renal epithelium; meanwhile, an apparent normal majority of renal parenchyma was seen (Fig. 6D).

### Immunohistochemical findings

**Fig. 7:**
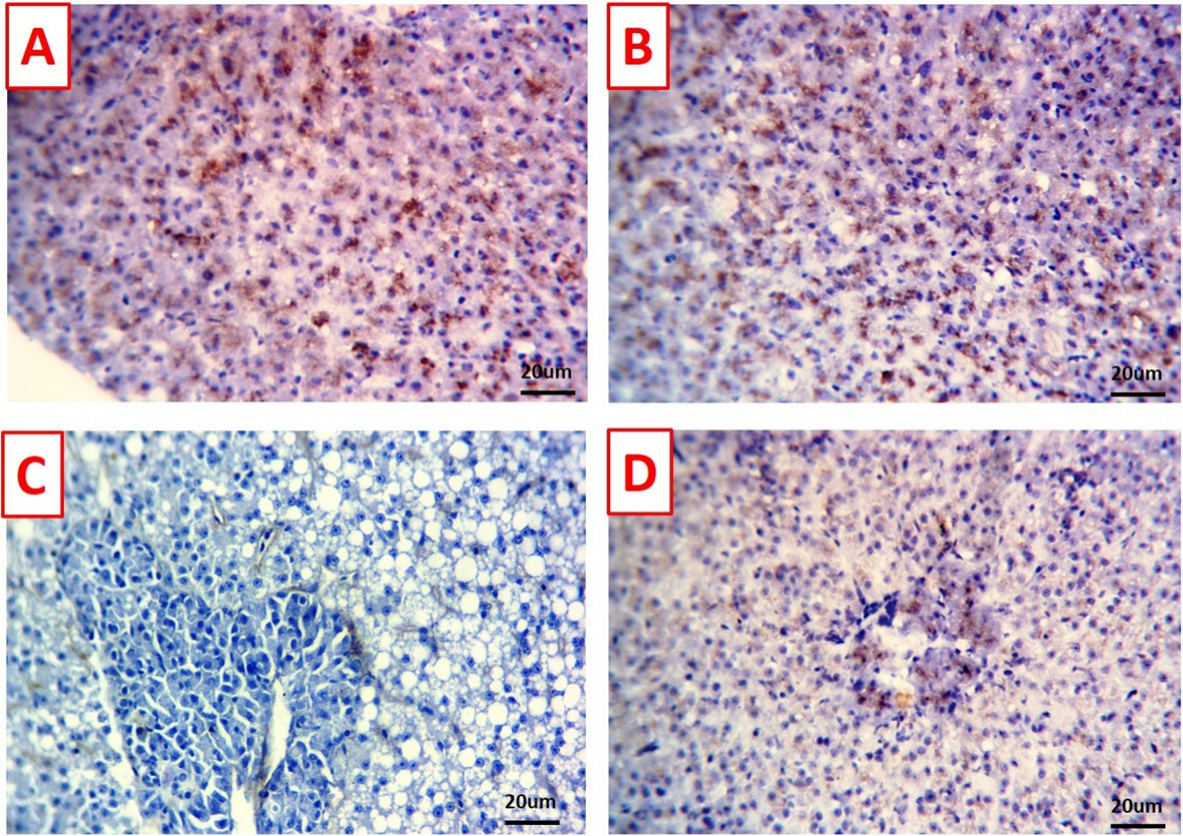
Photomicrographs of immunostained hepatopancreas sections (scale bar 20μm) for BCL-2 immunoreactivity. **A** & **B** Hepatopancreas of control and titanium dioxide nanogel (TDNG) groups displaying marked immune-staining level. **C** Hepatopancreas of *Pseudomonas putida* group displaying obvious reduction of immune-staining level. **D** Hepatopancreas of TDNG*+P. putida* group displaying mild to moderate immune-staining level

**Fig. 8:**
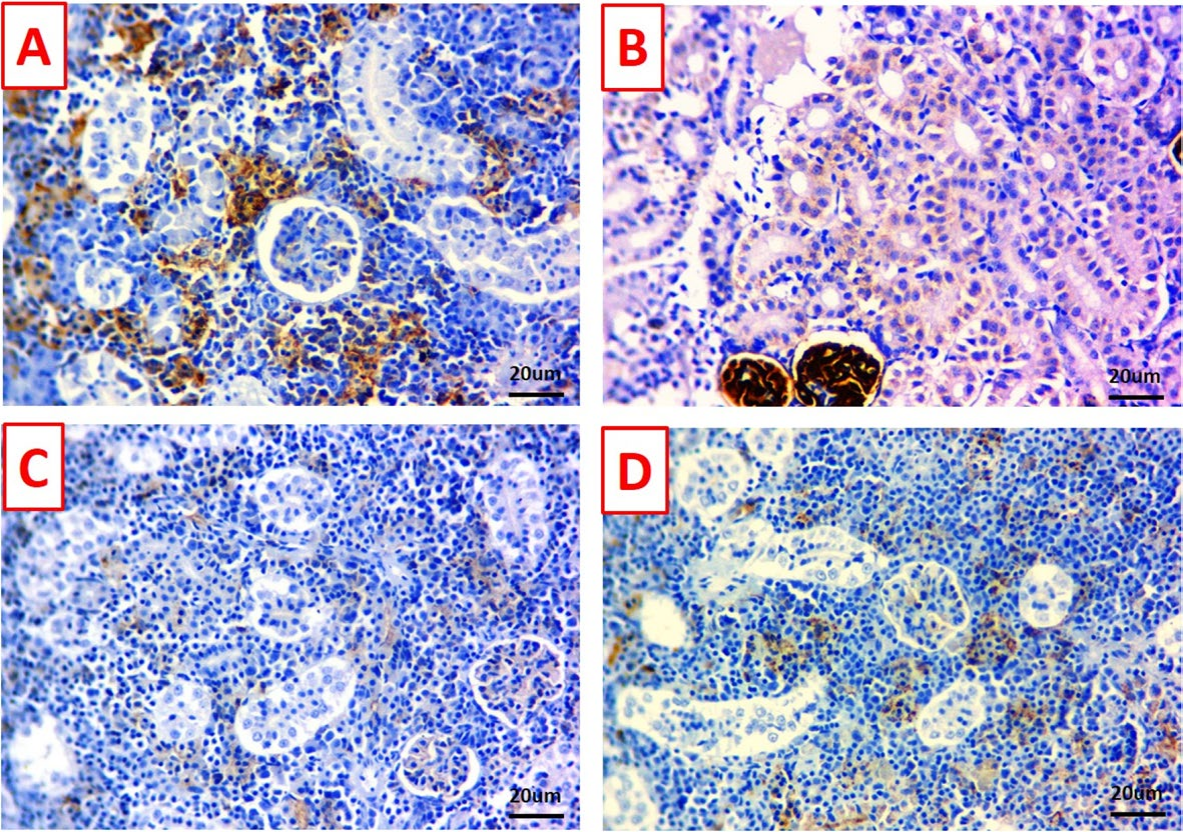
Photomicrographs of immunostained kidney sections (scale bar 20μm) for BCL-2 immunoreactivity. **A** & **B** Kidney of control and titanium dioxide nanogel (TDNG) groups display marked immune-staining level. **C** The kidney of the *Pseudomonas putida* group displays an obvious reduction of immune-staining level. **D** The kidney of TDNG*+P. putida* group displays mild to moderate immune-staining level

Immune-staining levels against BCL-2 appeared as brown granules in the hepatopancreas and kidney sections were markedly demonstrated in the control (Figs. 7A and 8A) and TDNG groups (Figs. 7B and 8B), respectively. *P. putida* infection induced an obvious reduction of BCL-2 expression in the hepatopancreas tissue (Fig. 7C) and negative expression in the kidney tissue (Fig. 8C). A mild to moderate number of immune-positive cells were obvious in the hepatopancreas (Fig. 7D) and kidney tissue (Fig. 8D) of the TDNG + *P. putida* group*.*

**Fig. 9:**
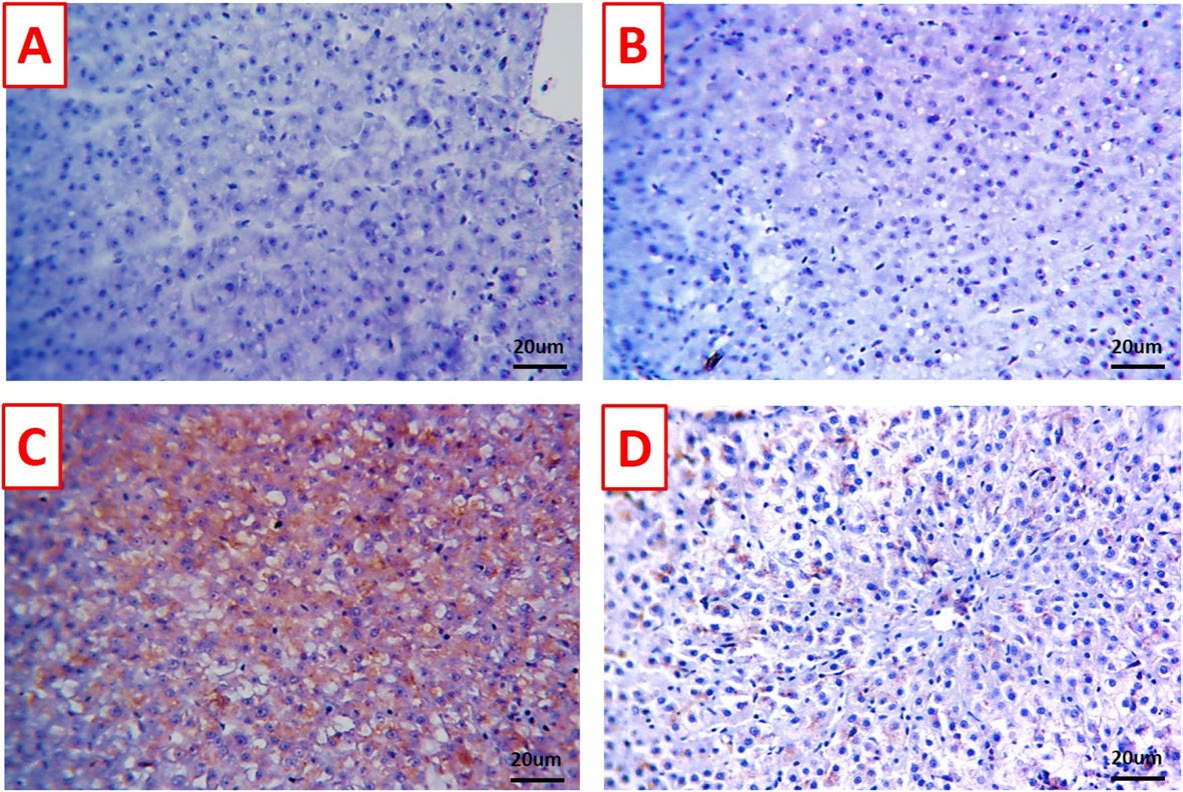
Photomicrographs of immunostained hepatopancreas sections (scale bar 20μm) for caspase-3 immunoreactivity. **A** & **B** Hepatopancreas of control and titanium dioxide nanogel (TDNG) groups display undetectable immunostained cells. **C** Hepatopancreas of the *Pseudomonas putida* group displays diffuse cytoplasmic expressions in a wide number of hepatic cells. **D** Hepatopancreas of TDNG*+P. putida* group displays few positive immunostained cells

**Fig. 10:**
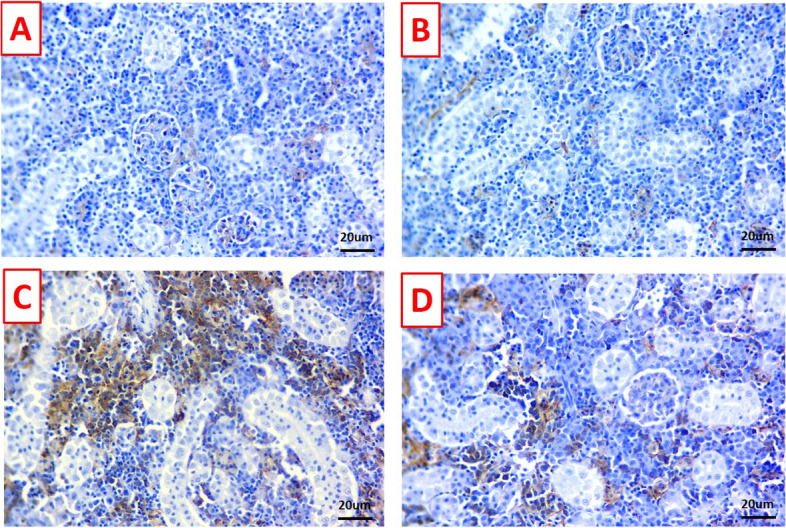
Photomicrographs of immunostained kidney sections (scale bar 20μm) for caspase-3 immunoreactivity. **A** & **B** The kidneys of the control and titanium dioxide nanogel (TDNG) groups display undetectable immunostained cells. **C** The kidney of the *Pseudomonas putida* group displays diffuse cytoplasmic expressions in a wide number of renal tubule cells. **D** The kidney of TDNG*+P. putida* group displays few number of positive immunostained cells

Figures 9 and 10 exhibited stained hepatopancreas and kidney sections against caspase-3, where undetectable immunostained cells in the control (Figs. 9A and 10A) and TDNG groups (Figs. 9B and 10B), respectively, were seen. Diffusely cytoplasmic expressions of caspase-3 in a wide number of hepatopancreas (Fig. 9C) and renal tubule cells (Fig. 10C) were observed in the *P. putida* group. Contrarily, the hepatopancreas (Fig. 9D) and kidney tissues (Fig. 10D) of the TDNG + *P. putida* group exhibited few positive immunostained cells.

## Discussion

Recent research has shown that applying nanomaterials in aquaculture fields enhances fish performance and health status [43, 44]. In the last decades, TiO_2_ NPs have been successfully applied as they own a proven toxic mechanism against bacteria [45]. Based on many studies, it is opined that metal oxides have positive charges. Meanwhile, the microorganism carries negative charges; this induces electromagnetic attraction between the metal oxides and the microorganisms, causing oxidization and death of microorganisms [46]. Therefore, the current perspective is based primarily on assessing the efficacy of aqueous exposure of TDNG on behavior, hepato-renal functions, immune-antioxidant capacity, histopathology, and immunohistochemistry in Nile tilapia experimentally infected with *P. putida*.

In evaluating the ethological changes, our study clarified that *P. putida* challenge caused various behavioral alterations (abnormal swimming, surfacing, resting, and aggression), clinical signs (skin darkness, body hemorrhages, and severe skin ulcerations), and lower survivability (56%). This could be dominated by the existence of virulence genes [exotoxin A (tox A), nan1, and the exo-enzyme S (exo S)], which hasten the infection process. Salama and Gharib [6] and Enany et al. [47] identified similar outcomes in Nile tilapia.

Exposure of the* P. putida*-infected fish to TDNG revealed a noticeable improvement in clinical signs and behaviors and a marked increase in fish survivability (84%). This could be dominated by the direct anti-bacterial action of nano-sized TDNG on the cell wall of *P. putida*. Earlier studies supported our findings and reported an interaction between the positive charge of TiO_2_ and the negative charges of the bacterial cells. Consequently, electromagnetic power was produced between metal oxide surfaces and bacterial cells. Moreover, TiO_2_ produces ions that can interact with the –SH group of proteins that limit the movement of material, lessening their permeability [45, 48].

C3 and LYZ have a substantial role in the innate immune function of fish against infection [49, 50]. Caspase-3 means extrinsic apoptosis which is a development of cell death that plays a crucial role in the homeostasis of tissues [51]. Meanwhile, intrinsic apoptosis is the response to a stimulatory reaction mediated by the interaction of the BCL-2 family and its membranes [52]. Interestingly, the current study displayed the immunosuppressing activity of *P. putida* expressed by a marked decline in the C3 and LYZ values, plus alterations in immuno-histopathological parameters (down-regulation of BCL-2 and strong caspase-3 immune reaction). This could be dominated by the direct action of the virulence genes which leads to cytotoxicity, and accordingly immune dysfunction [53]. Concurrently, Alzahrani et al. [9] revealed that *P. putida* inhibited the immune biomarkers. The basic attention is devoted to the immune-modulating activity of TDNG following exposure to the bacterial infection (*P. putida*) which is indicated by augmenting levels of C3 and LYZ plus up-regulation of the response of the BCL-2 and down-regulation of caspase-3. It is opined that TiO_2_ has a strong anti-bacterial action that can directly suppress bacterial activity by its nano-size [12, 54], inducing a remarkable improvement in the immune parameters and accordingly, strengthens the immune system.

The crucial antioxidant biomarkers, including TAC, SOD, and GSH, have a deep-rooted role in mitigating oxidative damage in the body via relapsing free radicals and reflecting antioxidant-defending activity. Oxidative stress is diligently associated with the incidence and progress of a disease, and when the body's antioxidant mechanism is unbalanced, it could produce oxidative damage in pathological states [55]. Therefore, it is essential to assess the antioxidant capacity, hepato-renal function, and histopathological alterations in response to exposure to NGs and bacterium to reflect the antioxidant status of the fish. The present study clarified the occurrence of oxidative damage in the hepatopancreas and kidneys in the infected group in *P. putida,* indicated by a reduction in the hepatic antioxidant biomarkers (TAC, SOD, and GSH), elevating hepatic and kidney function biomarkers (ALT, AST, and creatinine), and remarkable histopathological changes in hepato-renal tissues. The weakened antioxidant system and oxidative damage may be caused by increased reactive oxygen species (ROS) release in the cell membrane brought on by *P. putida* toxins [56]. Likewise, a recent study by Alzahrani et al. [9] documented that *P. putida* suppresses the activities of antioxidant indices (SOD and catalase).

However, the exposed group to TiO_2_ pronounced antioxidant and anti-bacterial activities indicated by an increase in the antioxidant parameters, a modulation in the hepato-renal biomarkers, and regeneration in the architecture in the hepato-renal tissues. An earlier study supported our findings and explained the antioxidant-antibacterial activity of TiO_2_ via inducing a sudden decrease in the integrity of the bacterial cell membrane plus ROS release where superoxide species is produced to degrade the biomolecules [57]. Similarly, Abdel Rahman et al. [23] reported a clear improvement in the architecture in the hepatic and renal tissues post-exposure of African catfish (*Clarias gariepinus*) to magnetite nanogel. Also, Mahboub et al. [24] displayed the strong antioxidant activity of chitosan nanogel indicated by modulating values of SOD and catalase.

## Conclusions

Based on the study outcomes, TDNG at a concentration of 0.9 mg/L is a versatile anti-bacterial tool against *P. putida* infection. It can decrease mortality, oxidative stress, and hepato-renal malfunction in *P. putida*-infected fish. Plus, it can be utilized as an immunomodulatory and antioxidant agent as it promotes activity on immune-antioxidant parameters and regenerates the histopathological changes induced by bacterial infection. Further studies are required to assess the dietary intervention of TDNG and test its influence on other fish species.

### Supplementary Information

**Supplementary Material 1.** 

## Data Availability

All data generated or analyzed during this study are included in this article.
