# Supplementary material for: Pseudomonas putida infection induces immune-antioxidant, hepato-renal, ethological, and histopathological/immunohistochemical disruptions in Oreochromis niloticus: the palliative role of titanium dioxide nanogel
Source: BMC Vet Res. 2024 Apr 1;20:127. doi: 10.1186/s12917-024-03972-6 (PMC10983678; doi:10.1186/s12917-024-03972-6)
Supplement: Supplementary file 1 — Supplementary Material 1. [file 12917_2024_3972_MOESM1_ESM.docx]

| **Supplementary Table 1** Effect of different titanium dioxide nanogel concentrations on clinical observation and mortality of Nile tilapia for ten days | | | | | |
| --- | --- | --- | --- | --- | --- |
| Conc. (mg/L) | Clinical observations | | |  | |
|  | Abnormal swimming | Loss of escape reflex | Skin lesions | | Mortality  (*n*=10) |
| 0.0 | - | - | - | | 0/10 |
| 0.3 | - | - | - | | 0/10 |
| 0.6 | - | - | - | | 0/10 |
| 0.9 | - | - | - | | 0/10 |
| 1.2 | - | + | - | | 0/10 |
| 1.5 | - | + | - | | 1/10 |
| 1.8 | ++ | ++ | - | | 2/10 |
| (+) Mild, (++) Moderate, and (− ) No signs or lesions | | | | | |
